# Ovarian tumor and glomerulopathies: case report and review of the literature

**DOI:** 10.11604/pamj.2019.34.75.6008

**Published:** 2019-10-05

**Authors:** Nawal Benabdellah, Hassan Izzedine, Yassamine Bentata, Intissar Haddiya

**Affiliations:** 1 Department of Nephrology, University Hospital Mohamed VI, Oujda, Morocco; 2 Department of Nephrology, Pitie-Salpetiere Hospital, 43 Boulevard de l'Hôpital, 75013 Paris, France

**Keywords:** Glomerulopathy, nephrotic syndrome, ovarian tumor

## Abstract

We describe a patient who developed nephrotic syndrome in the setting of ovarian tumor. A kidney biopsy showed minimal change nephropathy (MCN). CT scan and MR imaging followed by surgery lead to diagnostic of ovarian dermoid cyst. Surgery combined with corticosteroids resulted in a complete remission of nephrotic syndrome with disappearance of proteinuria after 3 weeks. Ten other cases of ovarian tumor associated with glomerulopathy are reviewed. This is the second case of an ovarian teratoma associated with MCN. Accurate history, physical examination, laboratory data, and kidney biopsy are highlighted in establishing the correct diagnosis in such patients.

## Introduction

The relationship between glomerulopathy and malignancy was first described in 1922 by Galloway [1] in a patient with Hodgkin's disease who developed massive proteinuria. This association was confirmed in 1966 by Lee *et al*. who found 11 patients with cancer in a group of 101 patients with a nephritic syndrome [2]. The most common renal pathology in these patients was membranous nephropathy with deposition of immune complex [3]. Minimal change nephropathy (MCN) is widely known to be associated with lymphoproliferative disorders, especially with Hodgkin's disease [4]. However, few cases of MCN have been reported in patients with solid tumors [5] and only in few previous reports of ovarian neoplasms associated with the nephrotic syndrome. Only one previously case of an ovarian teratoma associated MCN have been reported [6]. We herein describe a second case of such association.

## Patient and observation

A 55-year-old Philippine woman was admitted to our hospital, in December 2008 because of abdominal mass and generalized edema. Her medical history was otherwise unremarkable. No history of hypertension, diabetes mellitus, medications or illicit drug use was evident. Upon admission, physical examination revealed no significant abnormalities except for 3+ pitting edema on the lower extremities and a fluid wave on the abdomen, a blood pressure of 110/80 mmHg, and a pulse rate of 76 beats/min. Laboratory studies showed a total protein of 5.0 g/dl, a serum albumin of 0.9 g/dl, a total cholesterol of 365 mg/dl, and a serum creatinine of 0.7 mg/dl. Urinalysis revealed heavy proteinuria (4+) without significant casts. Twenty-four-hour urinary protein excretion was 20.0 g with a selectivity index of 0.07. Serum and urine immunoelectrophoresis results found no monoclonal protein. Complete blood count with blood glucose level, complement levels, immunological tests and viral serologies were within normal limits. Chest x-ray examination was also normal. Abdominal ultrasonographic and magnetic resonance imaging revealed a 10x10x11cm-sized, well-defined left ovarian cyst with a heterogeneous component suggesting an ovarian dermoid cyst. Tumor markers were normal (CA 125: Cancer Antigen 125 level,3,065 U/ml, CEA:Antigen carcino-embryonnaire 1.61 ng/ ml; CA 19-9: Cancer Antigen 19-9 (10.7 U/ml).

A percutaneous renal biopsy was performed. Light microscopy showed at least 25 normal glomeruli without proliferation neither focal segmental glomerulosclerosis lesions nor interstitial fibrosis. Tubules and vessels were unremarkable. No immunoglobulin or complement depositions were observed by immunofluorescence. Transmission electron microscopy was not performed. Based on these findings, a diagnosis of MCN was made. The patient underwent laparoscopic left salpingo-oophorectomy. Histopathology of the mass revealed a benign mature cystic teratoma with plenty of skin appendages, sebaceous glands, and mature adipose tissues. Post operatively, oral corticosteroid, at a dose of 1 mg/kg body weight (55 mg daily), and diuretic were started resulting in complete remission of the nephrotic syndrome within 3 weeks, with a serum albumin level of 3.8 g/dl and a 24-hour urinary protein excretion of 85 mg. The patient's renal function remained normal. Furthermore, a MEDLINE search was conducted to identify previous reports of nephrotic syndrome-associated ovarian tumor. The search was performed by intersecting ovarian tumor with nephrotic syndrome, proteinuria, carcinoma and teratoma. Identified articles were read carefully for references to other articles eventually not found by MEDLINE.

## Discussion

In our case, the absence of classic causes of nephrotic syndrome and the briskly response to surgical removal of the tumor, albeit with steroids suggests a relationship between MCN and ovarian teratoma. Nephrotic syndrome is a rare manifestation of malignancy associated paraneoplastic syndrome. Secondary MCN is defined by the presence of some identifiable extraglomerular disease process occurring concomitantly with the morphologic and functional abnormalities of MCN, irrespective of underlying pathogenetic mechanism [7]. The six categories of secondary MCN (mainly neoplastic) are listed in Table 1 [7]. Remission of paraneoplastic nephrotic syndrome was described at various times after the resection of tumors, which is connected with immunological dysregulation in the course of neoplastic disease [8]. In terms of ovarian malignancies associated with the NS, only few cases of ovarian tumor, mostly in ademocarcinoma type, presenting as nephrotic syndrome have been reported (Table 2) [2,6,9-19]. Nephrotic syndrome seems to occur irrespective to the ovarian tumor diagnostic time, either before (n=6, from 1 to 32 months), during a relapse 2 years after the first diagnosis (n=1), or simultaneously (n=5). The underlying glomerular lesions were membranous nephropathy (8 cases), membranoproliferative glomerulonephritis (2 cases), AA amyloidosis (2 cases), MCN (2 cases) and mesangial proliferative glomerulonephritis (1 case). A causative relationship between nephrotic syndrome and tumors would be indicated by the clinical remission of the nephrotic syndrome following the complete removal of the tumor without any additional therapy. But in some cases of nephrotic syndrome associated with ovarian tumors, the treatment has included the administration of corticosteroid with surgical removal of the tumor [2,13]. Surgery and/or chemotherapy and/or corticosteroids combination makes unclear the relationship between the remission of both tumor and nephrotic syndrome (Table 2).

**Table 1:** Renal histology in nephrotic syndrome associated with ovarian tumors

Authors	Age (yrs)	Onset of NS compared to discovery of tumor	Kidney histology	Ovarian tumor type	Treatment			Response of NS	Vital outcome at last follow up
					Surgery	Chemotherapy	Steroid		
Lee *et al*. [2]	65	18 months before	MN	Adenocarcinoma	No	No	No	No	Death
Lee *et al.* [2]	28	N/A	MN	Benign teratoma	Yes	No	Yes	No	N/A
Jeroudi *et al.*[6]	36	Simultaneously	MCN	Benign mature cyst teratoma	Yes	No	Yes	Yes	Alive
Hoyt *et al.* [9]	65	N/A	MN	Papillary serous carcinoma	Yes	Yes	N/A	Yes	Remission of the carcinoma
Torres *et al.* [10]	N/A	N/A	MN	Adenocarcinoma	Yes	N/A	Yes	N/A	N/A
Beauvais *et al.* [11]	7	10 months before	MN	Benign teratoma	Yes	No	No	Yes	Alive
Fernendez *et al.* [12]	64	12 months before	Amyloidosis	Poorly differentiated carcinoma	Yes	No	No	No	Death after surgery
Salazar *et al.* [13]	15	32 months before	MPGN	Mixed germ cells	Yes	No	Yes	No	No cured tumor; advanced RF
Forgy *et al.* [14]	68	1 month before	MN	Serous adenocarcinoma	Yes	Yes	No	Yes	Death with normal CA125 level
Topalak *et al.* [15]	65	6 months before	MN	N/A	N/A	Yes	N/A	Yes	Normalize CA125
Kim *et al.* [16]	59	Simultaneously	MPGN	Carcinoma	No	Yes	No	Yes	CR of the carcinoma
Ryu *et al.* [17]	55	Simultaneously	MCN	Papillary serous carcinoma	Yes	Yes	Yes	Yes	PR of the carcinoma
Ata *et al.* [18]	65	Two years after	MN	Clear cell carcinoma	Yes	Yes	Yes	Yes	Alive
Kilis-Pstrusinska *et al*. [19]	16	Simultaneously	MGN	Benign mature teratoma	Yes	No	Yes	Yes (delay)	Alive
This case	55	Simultaneously	MCN	Benign mature cyst teratoma	Yes	No	Yes	Yes	Alive

**Table 2:** Secondary minimal change disease: adapted from reference 7

**Neoplasia**	Hematological diseases( hodgkin’s disease, non-hodgkin’s lymphoma, leukaemia) Carcinoma (renal cell, eosophagus, bronchogenic, small cell lung, colon, pancreatic, prostatic, ovarien, renal oncocytoma) Others (thymoma, angiofollicular lymph node hyperplasia, mycosis fungoides, Neurilemmoma, chordoma, lymphoid hamartoma, kimura’s disease)
**Drugs**	Gold salts, antimicrobials, immunizations, lithium, interferon, tamoxifen, enalapril
**Infections**	Syphilis, tuberculosis, human immunodeficiency virus, mycoplasma, echinococcus
**Atopy**	Polen, dairy products (milk), house dust, pork, bee stings, poison oak/ ivy
**Superimposed on anotherrenal disease**	Ig A nephropathy, systemic lupus erythematous, HIV associated nephropathy, diabetes mellitus, autosomal dominant and recessive polycystic kidney disease
**miscellaneous**	Sclerosing cholangitis, sclerosing mesenteric inflammation, vigorous exercice, Acute decompression sickness, sarcoidosis, thyroiditis, vasculitis, partial Lipodystrophy, renal artery stenosis, bis-albuminaemia, Guillain-Barre syndrome, Dermatis herpetiformis, melorheostosis, mesenteric fibrosis

Remission of the NS was seen in 10 cases that achieved successful treatment of ovarian tumor. One patient obtained remission of nephrotic syndrome by excision alone [11], while remission is achieved with chemotherapy alone in another case [16] and by excision with chemotherapy in three other cases [9,14] On the other hand, remission of the NS was not achieved in two patients with ovarian tumor (mixed germ cells and dermoid cyst) treated with prednisone and excision [2,13] .Treatment and outcome could not be analyzed in the other cases due to incomplete information[10]. In our patient, surgical resection of the ovarian tumor, and corticosteroid treatment resulted in briskly and complete remission of the nephrotic syndrome. The possibility of a coincident occurrence of these conditions cannot be completely ruled out. However, considering the patient's age, the lack of other causes of nephrotic syndrome, the temporal relationship between diagnosis of cancer and onset of symptoms and the remission of the nephrotic syndrome following treatment of the ovarian cyst, this association does not seem to be fortuitous. The whole pathogenesis of secondary MCN has not been clearly defined, but a cell-mediated immune response has been postulated [20]; the secretion of a tumoral factor and/or the appropriate production of lymphokines by Tcells to suppress tumor growth could increase glomerular permeability [21]. Despite limitations in identifying a physical, mechanistic link between MCD (Secondary minimal change disease) and ovarian teratoma, future studies may lead to such findings.

## Conclusion

We describe the second case of an ovarian teratoma associated with MCN. Accurate history, physical examination, laboratory data, and kidney biopsy are highlighted in establishing the correct diagnosis. Surgery combined with corticosteroids resulted in a complete remission.

## Competing interests

The authors declare no competing interests.
